# Lessons learned from the safety investigation of an omitted dose-dispensed medicine

**DOI:** 10.1093/intqhc/mzag028

**Published:** 2026-03-04

**Authors:** Hanna Tiirinki, Alpo Vuorio

**Affiliations:** Department of Social Research, University of Turku, Turku, 20500, Finland; Safety Investigation Authority, Helsinki, 00520, Finland; Mehiläinen Airport Health Centre, Vantaa, 01530, Finland; Department of Forensic Medicine, University of Helsinki, Helsinki, 00014, Finland

## Abstract

**Graphical Abstract:**
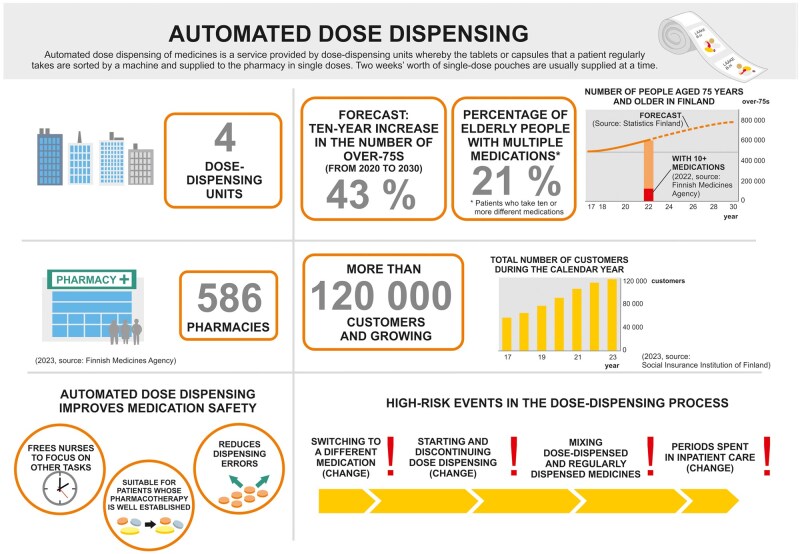

## Introduction

Issues related to automation in healthcare and its impact on safety and quality are continuously highly relevant topics [2]. Safety investigations in healthcare provide valuable lessons that help reduce similar incidents in the future [3]. Medication errors (MEs) have received widespread attention in recent decades and are a major concern for healthcare organizations worldwide [4]. Automated dose dispensing (ADD) is a technology-assisted system in which regularly used medicines are machine-packed in unit-dose bags according to administration times [5]. ADD is designed to improve medication safety and support medication management, especially for older home care clients and the elderly with polypharmacy [5–7]. A recent systematic review found that ADD can improve medication safety and quality of care [8]. However, despite its benefits, several types of MEs—such as prescribing errors—still persist. Medical errors can occur at any stage of the medication process, including prescribing, dispensing, administration, or medication follow-up. Dispensing is considered a high-risk activity, mainly due to the heavy workload faced by pharmacists. Although many of these errors are intercepted before reaching the patient, they remain a significant safety concern [6].

Emerging technologies also introduce new safety challenges, highlighting the need to better understand related risks. Another review emphasized that, due to the high costs and complex implementation of ADD, further studies on its cost-effectiveness are needed to guide decision-making [9].

There are several available ADD guidelines providing good practices [10–12]. The guidelines aim to improve ADD quality and reduce errors, encouraging error reporting. However, they lack practical instructions for investigating safety issues in the complex, multi-interface ADD system.

## Safety investigation of the omission of a dose-dispensed medicine

**Figure 1:**
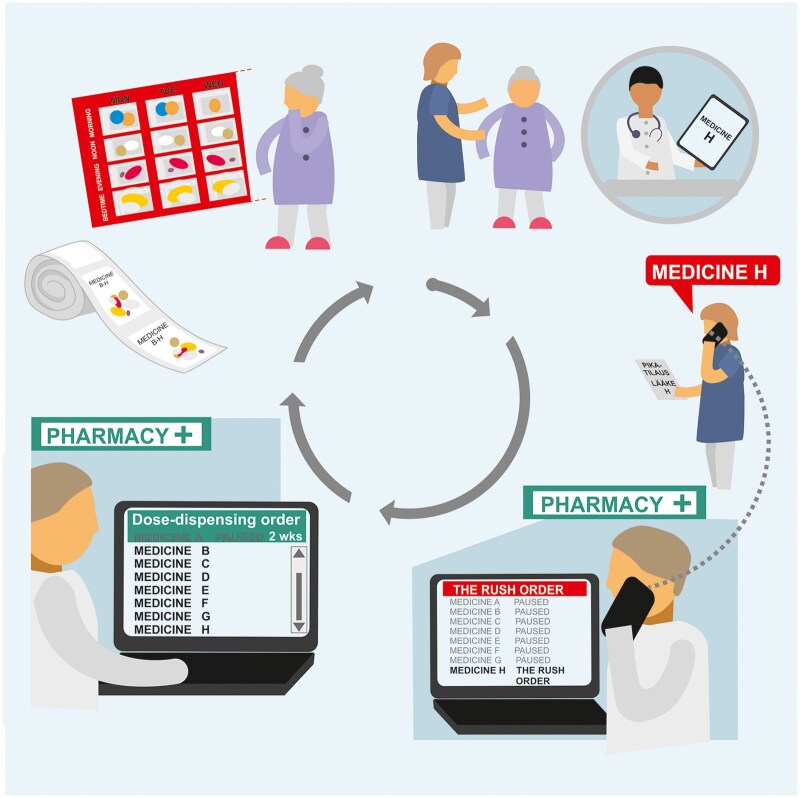
An automatic dose-dispensed medicine was omitted after the patient’s existing medications were paused in the system. The vertical list display made it unclear which medicines were paused [1].

The Finnish Safety Investigation Authority examined the omission of a medication in ADD [13]. In this incident, one prescribed medicine was omitted when the order was sent to the dose-dispensing unit (Fig. 1). The safety investigation was challenging due to ADD’s multiple interfaces with healthcare actors and regulators. Investigators also identified a need to explore interactions between healthcare professionals and the ADD system. The nearly year-long investigation included stakeholder hearings, a pharmacy staff survey, and reviews of both supervisory authorities’ work and the ADD system.

During the investigation [1], it was found that 586 pharmacies offer ADD services in Finland. The amount of dose-dispensing units is four. Currently, there are approximately 120,000 ADD customers, and the number of ADD customers is rapidly growing. At the moment, the percentage of elderly patients with multiple medications is estimated to be about 21% of all the ADD customers.

Based on the questionnaire addressed to pharmacists, the high-risk events in the ADD are related medication changes, starting and discontinuing ADD, mixing ADD and regularly dispensed medication and continuing ADD after the patient has visited the outpatient clinic.

The investigation highlighted risks related to ADD of medications, inconsistencies in operational practices, and gaps in legislation. Key national-level findings included a recommendation for the Ministry of Social Affairs and Health to assess the limitations of current information systems and consider legislative changes enabling more detailed regulatory requirements. The Ministry should also facilitate the effective communication of ME reports and lessons learned between pharmacies and well-being services counties, supported by clear procedures. The Finnish Medicines Agency was advised to strengthen pharmacy self-regulation in order to improve medication safety. Additionally, the Ministry should revise the *Guide on Good Practices in Patient-specific Dispensing of Medication* in collaboration with relevant stakeholders. At the international level, it was recommended to conduct comprehensive safety investigations into dose dispensing events across countries, with shared learning and harmonized development efforts that respect national legislation and practices.

## Conclusion

Dose dispensing is one example of a rapidly expanding process in healthcare, and its aim is to enhance both medication safety and cost efficiency [7, 8]. However, it is crucial to recognize the associated risks and allocate resources and time to understand the factors that may compromise patient safety. It is essential to identify and address these risks in ensuring the safe and effective implementation of such systems [3].

ADD of medications is increasingly utilized in healthcare, offering efficiency and safety benefits. However, emerging evidence highlights associated risks, including technical failures, medication selection errors, and insufficient system oversight. Integration challenges with existing healthcare workflows may lead to communication gaps and delays. Moreover, increased reliance on technology can diminish human oversight, potentially allowing errors to go unnoticed. A thorough understanding of these risks is critical in safeguarding patient safety.

## Data Availability

No new data were generated or analysed in support of this research.
